# Comparison of knee joint and temporomandibular joint development in pig embryos

**DOI:** 10.1080/10495398.2024.2337760

**Published:** 2024-04-24

**Authors:** Xiang Lei, Xuewen Wang, Yongfeng Li, Huawei Liu, Guoqiang Yan, Jinzhu Jing, Zhen Liang, Anyi Guo, Min Hu, Yajun Liu

**Affiliations:** a Beijing Research Institute of Traumatology and Orthopaedics, Beijing, PR China; b Beijing Jishuitan Hospital, Capital Medical University, Beijing, PR China; c Institute for Laboratory Animal Resources, National Institutes for Food and Drug Control, Beijing, PR China; d Department of Stomatology, Tsinghua Changgung Hospital, Beijing, PR China; e Department of Stomatology, the First Medical Center of PLA General Hospital, Beijing, PR China

**Keywords:** Pig, knee joint, temporomandibular joint, embryonic development, histology

## Abstract

Although the knee joint (KNJ) and temporomandibular joint (TMJ) all belong to the synovial joint, there are many differences in developmental origin, joint structure and articular cartilage type. Studies of joint development in embryos have been performed, mainly using poultry and rodents. However, KNJ and TMJ in poultry and rodents differ from those in humans in several ways. Very little work has been done on the embryonic development of KNJ and TMJ in large mammals. Several studies have shown that pigs are ideal animals for embryonic development research. Embryonic day 30 (E30), E35, E45, E55, E75, E90, Postnatal day 0 (P0) and Postnatal day 30 (P30) embryos/fetuses from the pigs were used for this study. The results showed that KNJ develops earlier than TMJ. Only one mesenchymal condensate of KNJ is formed on E30, while two mesenchymal condensates of TMJ are present on E35. All structures of KNJ and TMJ were formed on E45. The growth plate of KNJ begins to develop on E45 and becomes more pronounced from E55 to P30. From E75 to E90, more and more vascular-rich cartilage canals form in the cartilage regions of both joints. The cartilaginous canal of the TMJ divides the condyle into sections along the longitudinal axis of the condyle. This arrangement of cartilaginous canal was not found in the KNJ. The chondrification of KNJ precedes that of TMJ. Ossification of the knee condyle occurs gradually from the middle to the periphery, while that of the TMJ occurs gradually from the base of the mandibular condyle. In the KNJ, the ossification of the articular condyle is evident from P0 to P30, and the growth plate is completely formed on P30. In the TMJ, the cartilage layer of condyle becomes thinner from P0 to P30. There is no growth plate formation in TMJ during its entire development. There is no growth plate formation in the TMJ throughout its development. The condyle may be the developmental center of the TMJ. The chondrocytes and hypertrophic chondrocytes of the growth plate are densely arranged. The condylar chondrocytes of TMJ are scattered, while the hypertrophic chondrocytes are arranged. Embryonic development of KNJ and TMJ in pigs is an important bridge for translating the results of rodent studies to medical applications.

## Introduction

The knee joint (KNJ) and Temporomandibular joint (TMJ) are all belong to synovial joints, but there are many differences between them. In terms of joint developmental origin, the KNJ originates from the mesoderm, while the TMJ originates from ectodermal cranial neural crest.^1–3^ In terms of cartilage type, the cartilage of KNJ is primary cartilage, which belongs to hyaline cartilage, mainly Type II collagen and mucopolysaccharide. It is largely controlled by genetic factors and has little effect on the environment. It matures and secretes matrix through the division and proliferation of chondrocytes. However, the cartilage of TMJ is secondary cartilage and is characterized by Type I collagen. It is controlled not only by genetic factors, but also by more significant environmental factors. The condylar cartilage is gradually enlarged by the germinal cells beneath the perichondrium, and secretes the cartilage matrix after maturity. While some studies of the embryonic development of KNJ and TMJ have been reported, but the comparative development of these two joints has not been reported. What are the differences between KNJ and TMJ in features of spatio-temporal development and how articular cartilage develops into different types? These questions remain unclear. In human medicine, the study of morphological features and molecular regulatory mechanisms in the joint development of embryos is limited by sample size and the lack of continuity. Using model animals to simulate the development of human embryonic joints is a bridge to transform the results of animal research into human medicine. Previous studies of KNJ and TMJ have focused on rodents and poultry.^4–7^ However, the genetic relationship between poultry and humans is distant. There are great differences between rodents and humans. For example, the thickness of KNJ cartilage (average 30 mm) of mice is nearly 50 times thinner than that of human, the calcified cartilage layer of rodent KNJ is thicker than that of non-calcified cartilage layer, and chondrocytes lack obvious surface, transitional and radial areas.^8^ In TMJ, the absence of articular nodules and reduced function and smaller volume of the lateral pterygoid muscle in rodents. The superior articular cavity is first formed before the inferior, and the disk rarely changes to fibrocartilage with increasing age.^9^ These differences undoubtedly limit the application of rodents in joint study. Model animals more similar to human joints are an important part of joint-related research. It has been found that the morphology, proliferation, extracellular matrix synthesis and cytoskeletal assembly of pig joint are closer to those of humans.^10^ The arrangement of collagen in pig joint and human knee cartilage is similar, leaf-like, while in rats and mice it is columnar.^11^ There is no difference in mass or function of the cruciate ligament between humans and pigs. The KNJ main motion in pigs and humans is flexion/extension, while rotational motion is secondary. It has nothing to do with hoof movement or foot movement.^12^ Studies on the anatomical structure and embryonic development of TMJ in different animals have shown that the pig TMJ is similar to human in size and disk shape.^13–17^ However, a comparison of KNJ and TMJ development in large mammal-pigs during the embryonic stage has not been reported.

In this study, miniature pigs were used as experimental animals to compare the spatio-temporal development of KNJ and TMJ during the embryonic stage. It provides a basis for the pathogenesis of bone and cartilage-related diseases, regenerative medicine and tissue engineering.

## Materials and methods

This experiment was conducted according to the guidelines of the Ethical Review of Laboratory Animal Welfare (GB/T358922018). The study was approved and supervised by the Institutional Animal Care and Use Committee of Beijing Research Institute of Traumatology and Orthopedics (Beijing, China; approval document No. 2020-X16-109). Six sows, three newborn piglets (P0) and three piglets born 30 d after birth (P30) Bama miniature pigs were obtained from Beijing Kuibu Shichuang Biotechnology Company (license number, SCK2018-0011) for the study. The Bama miniature pig is a naturally generated strain in China. With the characteristics of small size, stable phenotypic traits and strong disease resistance, it is the most widely used miniature pig strain in the field of medical research in China. The pigs were separately fed in pens maintained under controlled conditions (temperature, 18–22 °C; relative air humidity, 40%–70%) and allowed free access to water. The KNJ and TMJ were harvested on embryonic day 30 (E30), E35, E45, E55, E75, E90, P0 and P30 from pregnant Bama miniature pigs and evaluated. The pigs were euthanized *via* bleeding under deep anesthesia with pentobarbital (3%). All samples were dissected by two surgeons using a surgical microscope to avoid tissue injury.

The E30, E35, E45, E55, E75 and E90 (*n* = 5 for each type) embryos were fixed in 10% formalin for 2 d. The E75, E90, P0 (*n* = 3) and P30 (*n* = 3) embryos and piglets of KNJ and TMJ were decalcified in 0.5 mol/L ethylenediaminetetraacetic acid solution (Solarbio Biological Company, Beijing, China) for 14 d and rinsed with tap water for 10 min. The KNJ and TMJ were removed from E45, E55, E75, E90, P1 and P30 to embed in paraffin for analysis. It was difficult to identify the joint structure in E30 and E35 embryo; therefore, the whole embryo was embedded in paraffin for evaluation. Semi-serial sagittal sections (3 μm) of the joint were prepared using a microtome (HM 325; Thermo Fisher Scientific, Waltham, MA). The samples were stained with Hematoxylin–Eosin (HE) to confirm overall tissue features and Safranin O-Fast green confirm chondrocyte development. HE staining was performed using a Leica AutoStainer XL (Leica Biosystems, Wetzlar, Germany). HE stain procedure is that tissue sections are dipped in xylene twice, each time for 10 min; Xylene and 100% ethanol mixture for 2 min; 100% ethanol for 5 min; wash 5 min; Hematoxylin dye for 5 min; wash 5 min; Hydrochloric acid ethanol differentiated solution for 1 s; 10 s for the ammonia-blue solution; washing 30 s; 2 min of Eosin dye solution; wash 30 s; 80% ethanol for 1 min; The 95% ethanol was immersed twice for 1 min each time, and the 100% ethanol was immersed twice for 3 min each time. Xylene was performed twice, each time for 3 min. The neutralized gum was sealed and observed under a light microscope. Safranin O-Fast Green stain procedure is that tissue sections are dewaxed, washed with water, stained with 1% saffranine O for 10 min, washed with 50%, 70% and 80% alcohol for 1 min each, stained with 0.5% Fast Green for 1 min and washed with 100% alcohol for 3 times, 5 s each time. The KF-view software was used to analyse the tissue sections.

## Result

### Anatomy of KNJ and TMJ

**Figure 1.**
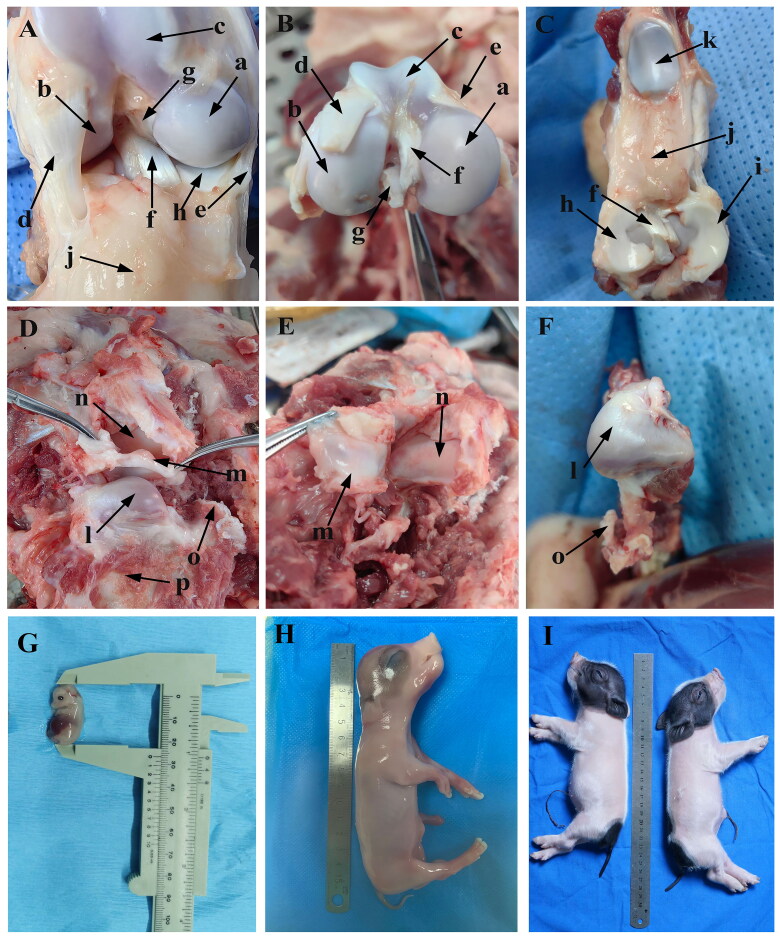
Anatomy of knee joint (KNJ) and temporomandibular joint (TMJ) in pigs. A–C: Anatomy of KNJ. a. Medial femoral condyle; b. Lateral femoral condyle; c. Femoral pulley; d. Lateral collateral ligament; e. Medial collateral ligament; f. Anterior cruciate ligament; g. Posterior cruciate ligament; h. Medial meniscus; i. Lateral meniscus; j. Subpatellar fat; k. Patella. D–F. Anatomy of TMJ. l. Mandibular condyle; m. Articular disk; n. Articular fossa; o. Coracoid process; p. Mandibular body; G. Embryo on E35; H. Embryo on E90; I. Embryo on P0

In order to better understand the structural characteristics of KNJ and TMJ in embryos, the KNJ and TMJ of adult miniature pigs were dissected in this study (Fig. 1). There is a great difference in the composition of KNJ and TMJ in pigs. The KNJ consists of the distal femur, proximal tibia, patella, cruciate ligament, meniscus, ligament and muscle. The KNJ does not have fossa like the TMJ (Fig. 1D, E, n). The femoral condyle of KNJ is divided into medial and lateral (Fig. 1A, B, a,b), while there is only one condyle in the TMJ (Fig. 1D, F, l). The TMJ articular disk (Fig. 1D, E, m) is thick at both ends and thin in the middle, and its function in the joint is similar to that of the KNJ. The TMJ articular disk is a complete articular cartilage, while the meniscus of KNJ is two parts of lateral thick and medial thin crescent-shaped cartilage (Fig. 1A, C, h,i). The pig embryos and piglets are shown in Fig. 1G–I.

### Histology of KNJ and TMJ in pig embryo

#### Observations on E30

**Figure 2.**
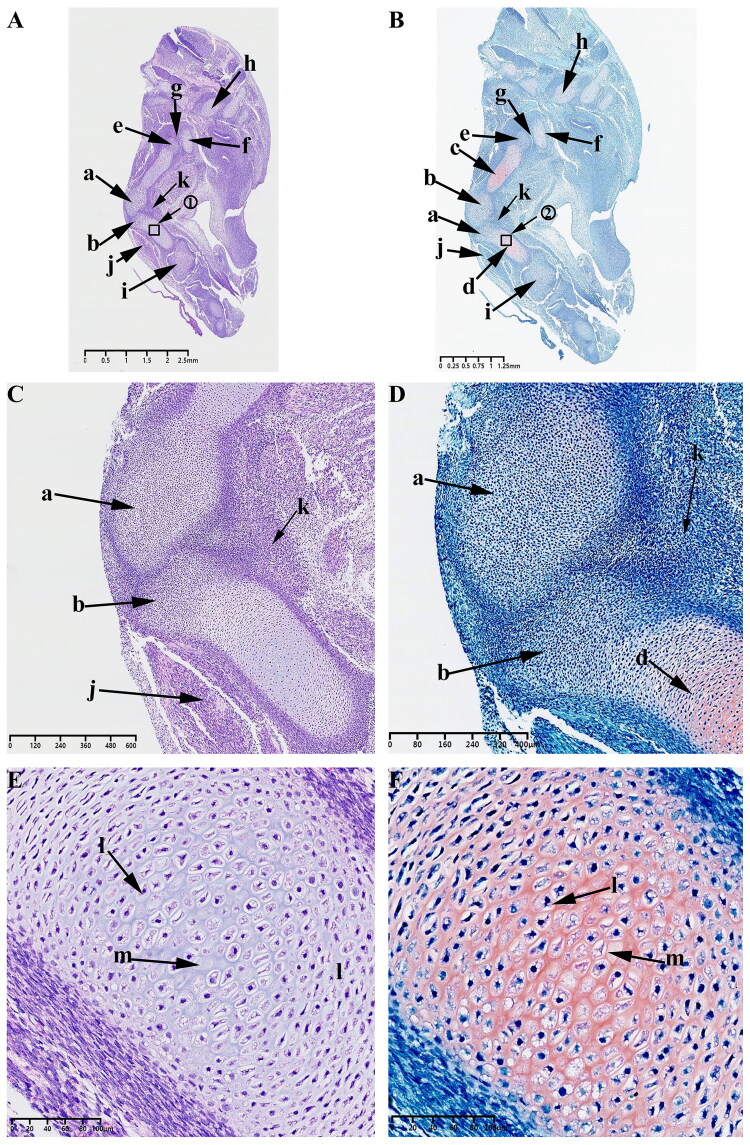
Sagittal histological section of Knee joint (KNJ) with hematoxylin–eosin and Safranin O-fast green staining on E30. The interzone and popliteal muscle mesenchymal condensate begin to appear in KNJ. Tibialis anterior muscle has been formed. The femur, tibial shaft and acetabulum begin to become cartilaginous. Chondrocytes are arranged in layers, and the cavitation cells can be seen in the tibia, femur and acetabulum. There is a long fissure between the head of the femur and the acetabulum. 1) magnify to E; 2) magnify to F. a,b. Mesenchymal condensate; c. Femoral shaft; d. Tibia shaft; e. Femoral head; f. Acetabulum; g. Articular cavity; h. Vertebra; i. Tarsal bone; j. Tibialis anterior muscle; k. Popliteal muscle mesenchymal condensate; l. Chondrocytes; m. Cavitating chondrocytes.

At this time, the typical developmental feature is the formation of mesenchymal condensates in the KNJ, which represents the beginning of joint development, while the TMJ has not yet begun to develop. HE and Safranin O-Fast green stains indicate the appearance of an interzone separating adjacent skeletal primordia on E30 in KNJ. This interzone is composed of two mesenchymal condensates (Fig. 2A, B, a,b). The interzone cells are high density and flat compared to the flanking cartilaginous skeletal anlagen containing round-shaped and more dispersed chondrocytes. These two mesenchymal condensates are not completely separated at this time. There are many mesenchymal cells connected to each other. The fusiform chondrocytes (Fig. 2E, F, l) which were stained red by saffron O have been found in the femur and tibia shaft (Fig. 2B, c,d). These chondrocytes were arranged in layers, and the cavitation cells (Fig. 2E, F, m) could be seen in tibia and femur shaft. There is a long fissure (Fig. 2A, B, g) between the head of the femur (Fig. 2A, B, e) and the acetabulum (Fig. 2D, f). Chondrocytes appear in the acetabulum (Fig. 2A,B, e). The tibialis anterior muscle (Fig. 2A–C, j) has been formed. Mesenchymal condensate (Fig. 2A–D, k) of popliteal muscle began to appear. No mesenchymal condensate of TMJ was found on E30. There was no sign of TMJ development on E30.

#### Observations on E35

At this time, the typical developmental feature is that there are two mesenchymal condensates are formed in TMJ, which represents the beginning of joint development.

**Figure 3.**
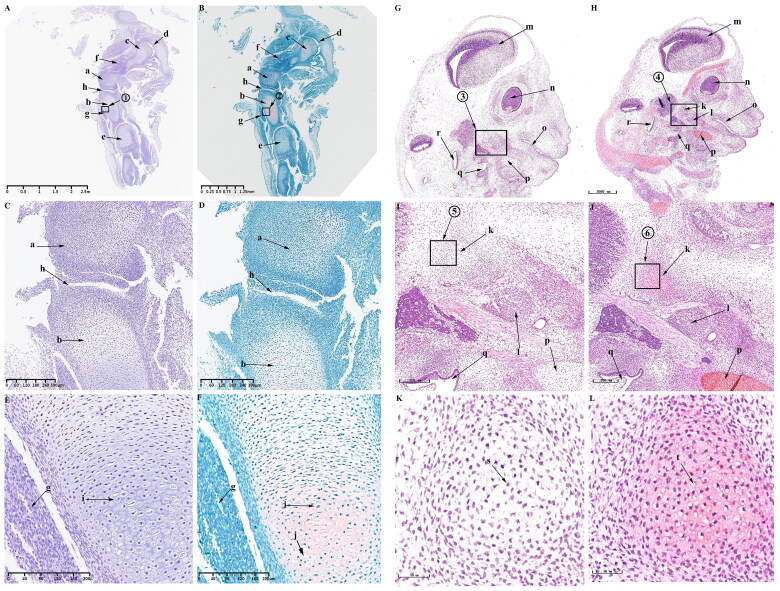
Sagittal histological section of Knee joint (KNJ) and Temporomandibular joint (TMJ) with hematoxylin–eosin and Safranin O-fast green staining on E35. The knee mesenchymal condensate is separated from the middle. The articular cavity is formed by cavitation between the femur and tibia. Chondrocytes surrounded by stem cells line the femur and tibia in a vortex. Cavitation occurs in some chondrocytes. Two mesenchymal condensates start to appear in the TMJ. Chondrification of the TMJ occurs in the temporal region. The cells line up in a vortex, and the chondrocytes in the center of the vortex begin to cavitate. There is no chondrification in the mandibular condyle. A–F. Histological analysis of KNJ; G–L. Histological analysis of TMJ. 1) magnify to E; 2) magnify to F; 3)magnify to I; 4) magnify to J; 5) magnify to K; 6) magnify to L. a. Femur; b. Tibia; c. Femoral head; d. Acetabulum; e. Tarsal bone; f. Lateral vastus muscle; g. Tibialis anterior muscle; h. Articular cavity; i. Chondrocytes; j. Cavitating chondrocytes; k. Temporal primordium; l. Condylar primordium; m. brain; n. eye; o. mouth; p. Meckel’s cartilage; q. Pharyngeal cavity; r. External auditory canal; s. Chondrocytes; t. Cavitating chondrocytes.

When the embryo reaches 35 d of development, the mesenchymal condensate of the KNJ separates and develops into the distal femur and proximal tibia (Fig. 3A–D, a,b).

The articular surface surrounding the synovial cavity remains rough and uneven. The mesenchymal cells of the femur and tibia are arranged in layers. The articular cavity (Fig. 3A–D, h) has formed by cavitation between femur and tibia. Chondrocytes surrounded by stem cells are arranged in a vortex (Fig. 3E, F, i) in the femur and tibia. Cavitation occurs in some chondrocytes (Fig. 3E, F, j). The femur and tibia shafts develop further cartilaginously. Femoral head (Fig. 3A, B, c) has formed and begun to become cartilaginous. The structures of hip joint cavity, acetabulum (Fig. 3A, B, d), lateral femoral muscle (Fig. 3A, B, f), tibialis anterior muscle (Fig. 3A, B, E, F, g) and tarsal bone (Fig. 3A, B, e) are clearly visible. During the development of TMJ, two mesenchymal condensates (Fig. 3G–J, k,l) start to appear, which are far apart from each other. These two mesenchymal condensates possibly develop into the condyle and temporal blastemas. An oval Meckel’s cartilage is found in the mandibular region (Fig. 3G–J, p). Chondrification of the TMJ occurs first in the temporal region (Fig. 3G–J, k). In this region, the cells line up in a vortex, and the chondrocytes (Fig. 3K, L, s) in the center of the vortex begin to cavitate (Fig. 3L, t). The brain (Fig. 3G, H, m), eyes (Fig. 3G, H, n), mouth (Fig. 3G, H, o), pharynx (Fig. 3G–J, q), external auditory canal (Fig. 3G, H, r) and other structures are clearly visible.

#### Observations on E45

**Figure 4.**
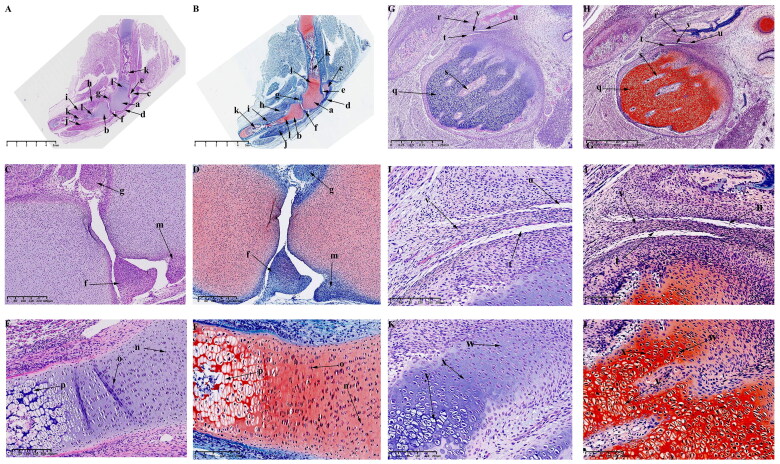
Sagittal histological section of KNJ and TMJ with hematoxylin–eosin and Safranin O-fast green staining on E45. The meniscus and articular eminence are clearly visible. The patella develops and begins to become cartilaginous. There is a concentrated zone of chondrocytes which develop into epiphyseal plates below the chondrocyte layer of the femur and tibia, and the cells are fusiform. The superior and inferior articular cavities of the TMJ have been formed. There is still fibrous tissue in the superior and inferior articular cavities. A–F. Histological analysis of KNJ; G–L. Histological analysis of TMJ. a. Femoral condyle; b. Tibial plateau; c. Patella; d. Patellar ligament; e. suprapatellar bursa; f. Meniscus; g. Eminence of medial joint; h. Soleus muscle; i. Popliteal muscle; j. Tibialis anterior muscle; k. Mineralized area; l. Growth plate; m. Eminence of lateral joint; n. Chondrocytes; o. Growth plate; p. Hypertrophic chondrocytes; q. Condyle; r. Temporal bone; s. Cartilage canal; t. Inferior articular cavity; u. Superior articular cavity; v. Articular disk; w. Round chondrocytes; x. Fusiform chondrocytes y. Hypertrophic chondrocytes.

At this time, the obvious developmental feature is the formation of the main structures of KNJ and TMJ. The growth plate of the KNJ begins to appear. After 45 d of embryonic development, all structures of the KNJ have developed. The femur (Fig. 4A, B, a) and tibia (Fig. 4A, B, b) are filled with a large number of chondrocytes. The patella (Fig. 4A, B, c) develops and begins to become cartilaginous. The patellar ligament (Fig. 4A, B, d) extends from the bottom of femoral condyle to the tibia. The development of suprapatellar bursa (Fig. 4A, B, e) is complete. The meniscus (Fig. 4A–D, f) and articular eminence (Fig. 4A–D, g,m) are clearly visible. The popliteal (Fig. 4A, B, h), soleus (Fig. 4A, B, i) and tibialis anterior muscles (Fig. 4A, B, j) are attached to the tibia. At this time, the femur and tibia are mineralized, and a large amount of bone tissue (Fig. 4A, B, k) is formed in the middle portion of the femur and tibia. There is a concentrated zone of chondrocytes (Fig. 4A, B, l) below the chondrocyte layer of the femur and tibia, and the cells are fusiform (Fig. 4E, F, o). These chondrocytes will develop into epiphyseal plates. There are obvious differences in morphology between growth plate cells (Fig. 4E, F, o) and chondrocytes (Fig. 4E, F, n). During the development of TMJ, multiple medullary cavities (Fig. 4G, H, s) surrounded by a large number of chondrocytes are seen in the mandibular condyle (Fig. 4G, H, q). The temporal bone (Fig. 4G, H, r) begins to ossify. The superior and inferior articular cavities (Fig. 4G–J, u,t) have been formed. There is still fibrous tissue in the superior and inferior articular cavities.The articular disk (Fig. 4G–J, v) begins to show the typical features of being thick at both ends and thin in the middle. The mandibular condyle cells are mainly composed of circular chondrocytes (Fig. 4K, L, w), fusiform chondrocytes (Fig. 4K, L, x) and hypertrophy and cavitation chondrocytes (Fig. 4K, L, y).

#### Observations on E55

**Figure 5.**
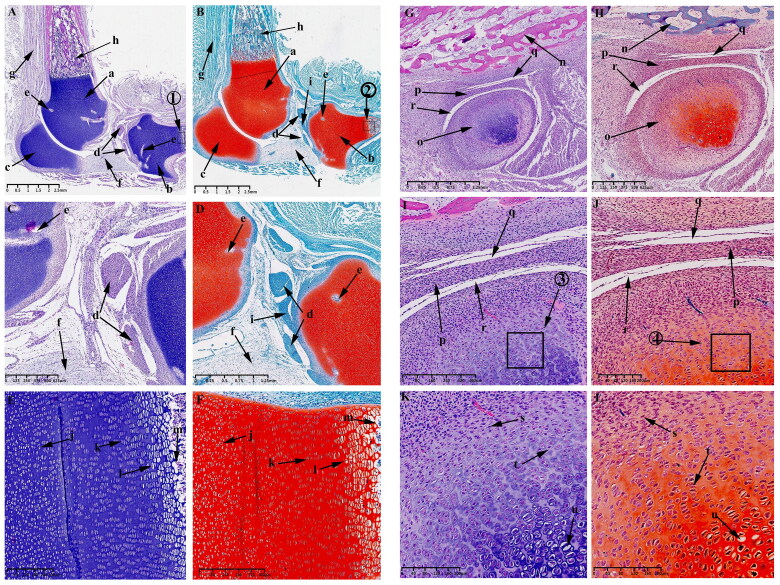
Sagittal histological section of KNJ and TMJ with hematoxylin–eosin and Safranin O-fast green staining on E55. The tibia and femur are still rich in chondrocytes, forming vascular-rich cartilage canals in the group of chondrocytes to provide nutrition for chondrocytes. During the development of TMJ, the temporal bone is completely ossified. The mandibular condyle is typically oval. The articular disk, superior articular cavity and inferior articular cavity have been formed. A–F. Histological analysis of KNJ; G–L. Histological analysis of TMJ. 1) magnify to E; 2) magnify to F; 3) magnify to K; 4) magnify to L. a. Femur; b. Tibia; c. Patella; d. Meniscus; e. Cartilage canal; f. Infrapatellar fat pad; g. Tendon of quadriceps femoris; h. Mineralized area; i. Anterior cruciate ligament; j. Oval chondrocytes; k. Round chondrocytes; l. Hypertrophic chondrocytes; m. Mineralized area; n. Temporal bone; o. Mandibular condyle; p. Articular disk; q. superior articular cavity; r. inferior articular cavity; s. Fusiform chondrocyte; t. Oval chondrocytes; u. Hypertrophic chondrocytes.

At this time, the KNJ and TMJ are further developed, and many cartilage canals are formed in the femoral and tibial condyles of the KNJ.The tibia (Fig. 5A, B, b) and femur (Fig. 5A, B, a) are still rich in chondrocytes, forming vascular-rich cartilage canal (Fig. 5A–D, e) in the group of chondrocytes to provide nutrition for chondrocytes. The patella (Fig. 5A, B, c) is rich in chondrocytes, the subpatellar fat pad (Fig. 5A–D, f) is formed and the tendon of quadriceps femoris (Fig. 5A, B, g) is more clearly visible. There is no cartilage canal formed in patella. The joint space continues to increase. The meniscus (Fig. 5A–D, d) and cruciate ligament (Fig. 5B, D, i) has been developed. The arrangement of the chondrocytes in the femur and tibia is the same. Along the long axis of the tibia, chondrocytes can be divided into three layers from top to bottom, which are round-shaped, irregularly arranged chondrocytes (Fig. 5E, F, j), fusiform chondrocytes, rows of closely arranged chondrocytes (Fig. 5E, F, k) and large hypertrophic chondrocytes with cavitation (Fig. 5E, F, l). Further ossification (Fig. 5E, F, m) of the tibia and femur occurs at 55 d of embryonic development. During the development of TMJ, the temporal bone is completely ossified (Fig. 5G, H, n). The mandibular condyle is typically oval (Fig. 5A, B, o). The articular disk (Fig. 5G–J, p), superior articular cavity (Fig. 5G–J, q) and inferior articular cavity (Fig. 5G–J, r) have been formed. There are still a few fibers in the superior and inferior articular cavity. Condylar chondrocytes include fusiform chondrocytes (Fig. 5K, L, s), round chondrocytes (Fig. 5K, L, t) and hypertrophic chondrocytes (Fig. 5K, L, u).

#### Observations on E75 and E90

**Figure 6.**
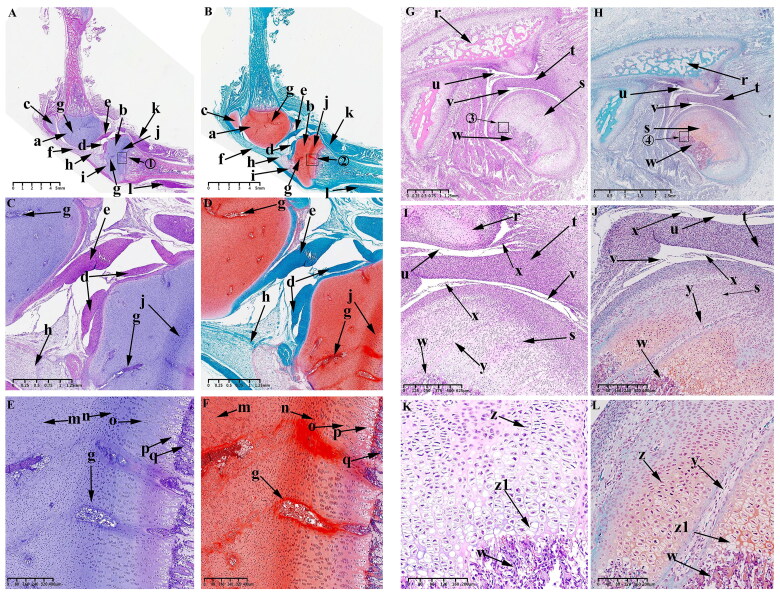
Sagittal histological section of knee joint (KNJ) and temporomandibular joint (TMJ) with hematoxylin–eosin and Safranin O-fast green staining on E75. The meniscus, cruciate ligament, infrapatellar fat pad and intercondylar process are clearly visible. The growth plate is more obvious. There are four chondrocytes types in the growth plate. The chondrocytes above the growth plate are rounded and irregularly arranged. The chondrocytes of the second layer are tightly packed in clusters, and those of the third layer are closely aligned in rows. The lowest-lying chondrocytes have hypertrophy and cavitation. Mineralization occurs below the condylar cartilage layer in the TMJ. The cartilage canal in TMJ extends along the longitudinal axis of the condyle, dividing the condyle into several parts, while the cartilage canal in the KNJ is arranged vertically and horizontally. A–F. Histological analysis of KNJ; G–L. Histological analysis of TMJ. 1) magnify to E; 2) magnify to F; 3) magnify to K; 4) magnify to L. a. Femur; b. Tibia; c. Patella; d. Meniscus; e. Anterior cruciate ligament; f. Patellar ligament; g. Cartilage canal; h. Infrapatellar fat pad; i. Intercondylar eminence; j. Growth plate; k. Popliteal muscle; l. Tibialis anterior muscle; m. Round chondrocytes; n. Clusters of chondrocytes; o. Columnar arranged chondrocytes; p. Hypertrophic chondrocytes; q. Mineralized area; r. Temporal bone; s. Mandibular condyle; t. Articular disk; u. superior articular cavity; v. inferior articular cavity; w. Mineralized area; x. Fibrous tissue; y. Cartilage canal; z. Chondrocytes; z1. Hypertrophy chondrocyte.

**Figure 7.**
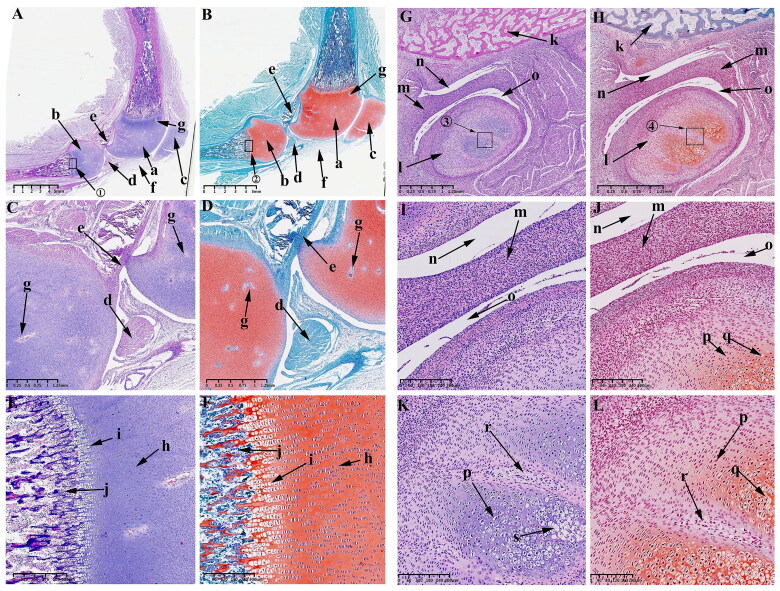
Sagittal histological section of knee joint (KNJ) and temporomandibular joint (TMJ) with hematoxylin–eosin and Safranin O-fast green staining on E90. The 90th day of embryonic development is the further maturation stage of KNJ and TMJ. The development of KNJ and TMJ is similar to that of E55. The most pronounced feature on E90 is the production of more cartilage canals in each chondrocyte region. A–F. Histological analysis of KNJ; G–L. Histological analysis of TMJ. 1)magnify to E; 2)magnify to F; 3)magnify to K; 4)magnify to L. a. Femur; b. Tibia; c. Patella; d. Meniscus; e. Cruciate ligament; f. Infrapatellar fat pad; g. Cartilage canal; h. Chondrocytes; i. Chondrocyte hypertrophy; j. Mineralized area; k. Temporal bone; l. Mandibular condyle; m. Articular disk; n. superior articular cavity; o. inferior articular cavity; p. Chondrocytes; q. Hypertrophy chondrocyte; r. Cartilage canal.

From E75 to E90, the growth plate is more pronounced in the KNJ, but there is still no growth plate formation in TMJ, and the cartilage canals begin to appear in the mandibular condyle. When embryo develops to 75 d, the femoral (Fig. 6A, B, a), tibial condyle (Fig. 6A, B, b) and patella (Fig. 6A, B, c) are still rich in chondrocytes. The lateral and medial corner of the meniscus (Fig. 6A–D, d) is close to the tibial plateau. The cruciate ligament (Fig. 6A–D, e) connects femoral condyle and tibial. The infrapatellar ligament (Fig. 6A–D, f) connects the patella to the tibia. Many cartilage canals (Fig. 6A–F, g) are seen in femoral condyle, tibial and patella. The infrapatellar fat pad (Fig. 6A–F, h) is located on the anterior side of the tibial plateau and connected to the infrapatellar ligament. The intercondylar process (Fig. 6A, B, i) is clearly visible. The tibia and femur have a dark stained band (Fig. 6A, B, j) of cells below the chondrocyte region that will develop into a growth plate in the future. The popliteus muscle (Fig. 6A, B, k) and tibialis anterior muscle (Fig. 6A, B, l) are tightly attached to the posterior and anterior side of the tibia, respectively. After enlarging the region near the tibial growth plate, it was found that there are four main chondrocytes types in this region. The chondrocytes (Fig. 6E, F, m) above the growth plate are rounded and irregularly arranged; the chondrocytes (Fig. 6E, F, n) of the growth plate are closely arranged in clusters, the chondrocytes (Fig. 6E, F, o) under the growth plate are closely arranged in rows, and the lowest chondrocytes (Fig. 6E, F, p) has hypertrophy and cavitation. The distal femur and proximal tibia has been mineralized (Fig. 6E, F, q). The TMJ on 75 d of embryonic development is similar to E55. The temporal bone (Fig. 6G–1, r) is completely ossified. Mineralization (Fig. 6G–L, w) occurs below the condylar cartilage layer. There is still fibrous tissue (Fig. 6G–J, x) in the superior (Fig. 6G–J, u) and inferior cavities (Fig. 6G–J, v). The cartilage canal (Fig. 6I–L, y) in the TMJ extends along the longitudinal axis of the condyle, dividing the condyle into several parts, while the cartilage canal (Fig. 6A–F, g) in the KNJ is arranged vertically and horizontally. From the 75th to the 90th day of embryonic development is the further maturation stage of KNJ and TMJ. The most pronounced feature on E75 and E90 is the production of more cartilage canals (Fig. 7A–D, g; Fig. 7G–L, r) in each chondrocyte region to provide nutrients to the chondrocytes.

#### Observations on P0 and P30

**Figure 8.**
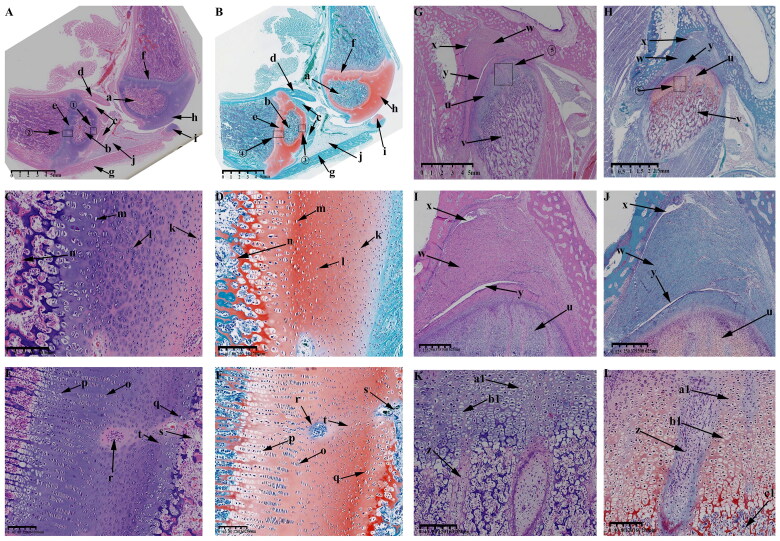
Sagittal histological section of Knee joint (KNJ) and temporomandibular joint (TMJ) with hematoxylin–eosin and Safranin O-fast green staining on P0. The secondary ossification center of KNJ are established in the epiphysis of the femur and tibia. There was no secondary ossification center in the mandibular condyle. A–F. Histological analysis of KNJ; G–L. Histological analysis of TMJ. 1) magnify to C; 2) magnify to E; 3) magnify to D; 4) magnify to F; 5) magnify to K; 6) magnify to L. a. Femoral epiphysis; b. Tibia epiphysis; c. Meniscus; d. Cruciate ligament; e, f. Growth plate; g. Patellar ligament; h. Articular surface cartilage; i. Patella; j. Infrapatellar fat; k. Prechondral cell; l. Chondrocytes; m. Hypertrophic chondrocytes; n. Mineralized area under the growth plate; o. Growth plate chondrocytes; p. Growth plate chondrocyte hypertrophy; q. Hypertrophy chondrocytes on the joint surface; r. Bone marrow cavity; s. Mineralized area above the growth plate; t. chondrocytes on the joint surface u. Mandibular condyle; v. Mineralized area; w. Articular disk; x. Superior articular cavity; y. inferior articular cavity; z. Bone marrow cavity; a1, Chondrocytes; b1, Hypertrophic chondrocytes; c1, Mineralized area.

**Figure 9.**
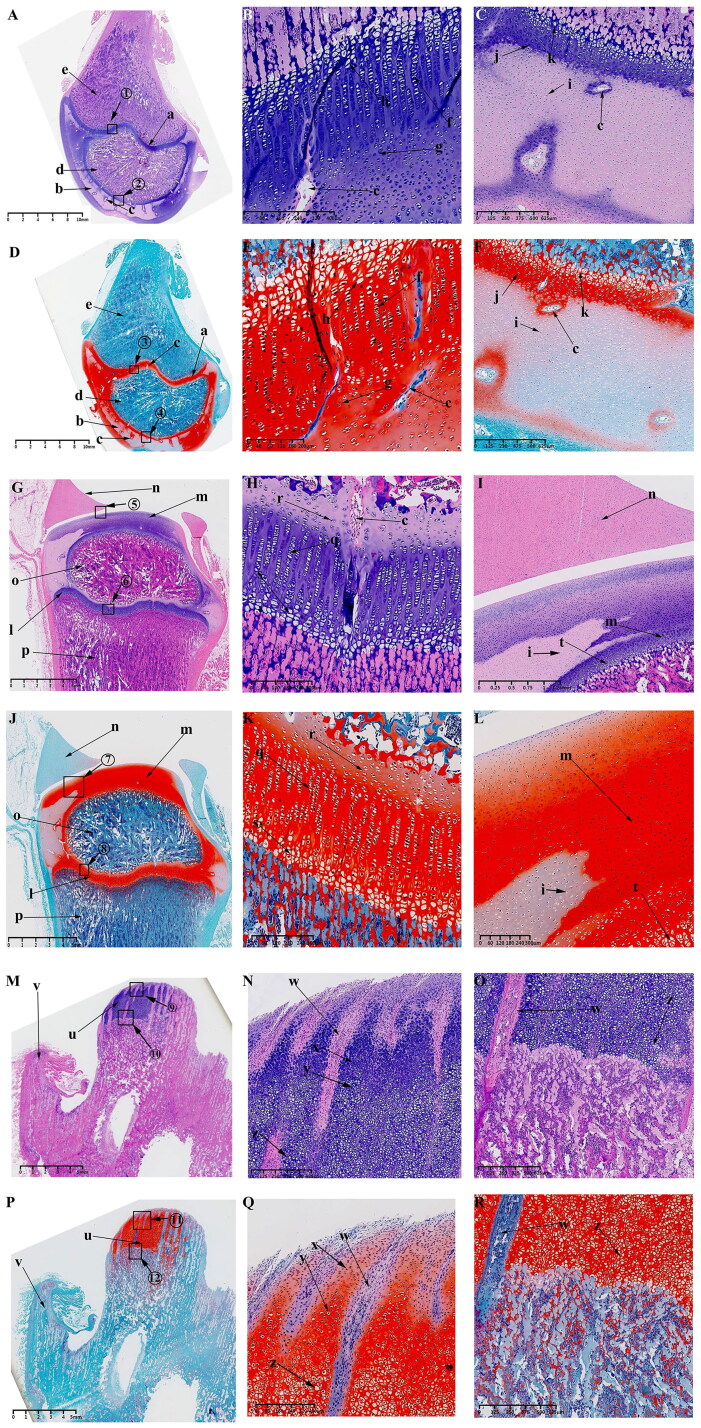
Sagittal histological section of Knee joint (KNJ) and Temporomandibular joint (TMJ) with hematoxylin–eosin and Safranin O-fast green staining on P30. The growth plate is still attached to the femoral condylar cartilage at the edges. Chondrocytes of the growth plate are densely packed and arranged along the longitudinal axis of the femoral and tibia condyles. In the development of TMJ, the chondrocyte layer of the mandibular condyle becomes thinner. The condyle is divided into several parts by multiple cartilage canals. There is no growth plate formation in TMJ. A-F. Histological analysis of epiphysis; G–L. Histological analysis of proximal tibia; M–R. Histological analysis of mandibular condyle. 1) magnify to B; 2) magnify to C; 3) magnify to E; 4) magnify to F; 5) magnify to I; 6) magnify to H; 7) magnify to L; 8) magnify to K; 9) magnify to N; 10) magnify to O; 11) magnify to Q; 12) magnify to R. a. Growth plate; b. Chondrocyte layer of femoral condyle; c. Cartilage canal; d. Ossification area of femoral condyle; e. Ossification area of femoral shaft; f. Growth plate chondrocytes arranged in rows; g. Growth plate chondrocytes; h. Hypertrophic chondrocytes; i. Surface cells of femoral condyle; j. Chondrocytes of femoral condyle; k. Hypertrophic chondrocytes of femoral condyle; l. The growth plate of the tibia; m. Surface cell layer of tibia; n. Meniscus; o. Ossification area of tibial condyle; p. Ossification area of tibial shaft; q. Chondrocytes of tibial growth plate; r. Chondrocytes of tibial condyle; s. Hypertrophic chondrocytes of tibial growth plate; t. Hypertrophic chondrocytes of tibia; u. Mandibular condyle; v. Coracoid process of mandible; w. Cartilage canal; x. prechondrocytes; y. Condylar chondrocytes; z. Hypertrophic chondrocytes.

From P0 to P30, the femoral and tibial condyles of the KNJ ossified from the middle to the periphery, while the TMJ is gradually ossified from the base of the condylar cartilage and the cartilaginous layer is gradually thinned. The secondary ossification center of KNJ is established in the epiphysis of the femur and tibia (Fig. 8A, a,b) on P0. Ossification of the femoral condyle and tibial condyles begins to occur from the center to the perimeter. The range of ossification gradually widens from P0 to P30, until P30 is almost complete (Fig. 9A, D, G, J, d,o) with only the cartilage at the edge of the joint surface connected to the growth plate.

The surface chondrocyte layer (Fig. 9A, D, b; Fig. 9G, J, m) of femoral and tibia condyles connected to the growth plate on P30 is thinner than that of P0. In the development of TMJ, the mandibular condylar cartilage layer is gradually thinned from P0 to P30. Other characteristics of P0 and P30 are generally consistent. At 30 d after birth, the growth plates of femur (Fig. 9A, D, a) and tibia (Fig. 9G, J, l) have been fully developed. A large amount of ossified tissue was produced in the femoral (Fig. 9A, D, d) and tibia condyles (Fig. 9G, J, o). The round cells (Fig. 9F, 1, L, i) on the joint surface become smaller and are not easily stained by saffron O. Chondrocytes (Fig. 9C, F, 1, L, j,m) and hypertrophic chondrocytes (Fig. 9C, F, 1, L, k, t) can be seen in the upper part of the mineralized area near the joint surface. There are a large number of cartilage canals (Fig. 9A–H, c) in the cartilage tissue of femoral condyle and growth plate. Unsaturated chondrocytes in the upper part of the growth plate (Fig. 9B, E, g; Fig. 9H, K, r) are round and loosely arranged. The growth plate chondrocytes (Fig. 9B, E, f; Fig. 9H, K, q) are arranged densely and arranged along the longitudinal axis of the femoral and tibia condyle. The osteoblasts between the growth plate and the surface of the femoral and tibia condyle (Fig. 9A, D, d; Fig. 9G, J, o) are arranged in clusters, and the osteoblasts (Fig. 9A, D, e; Fig. 9G, J, p) under the growth plate are arranged in rows. In the development of TMJ, the chondrocyte layer (Fig. 9M, P, u) of mandibular condyle becomes thinner. No chondrocyte formation was found in coracoid process (Fig. 9M, P, v). The condyle contains prechondrocytes (Fig. 9N, Q, x), chondrocytes (Fig. 9N, Q, y) and hypertrophic cartilage (Fig. 9N, Q, z). The condyle is segmented several parts by multiple cartilage canals (Fig. 9N, Q, w). A large number of mineralized tissues can be seen under the condylar cartilage.

## Discussion

Both KNJ and TMJ belong to the synovial joint, but its articular cartilage belongs to the hyaline cartilage and fibrocartilage, respectively. The joint diseases, such as osteoarthritis (OA) can occur in both joints. OA caused by degenerative condition, affects 3.8% of the world’s population and affects more than 40% of people over the age of 70.^18^^,^^19^ Among all kinds of diseases related to KNJ, the arthritis of the knee affects one in three people over the age of 40 in Western countries, patellar injury accounts for 1% of bone injury, meniscus injury with osteoporosis in elderly patients accounts for 30–40% and the incidence in high-load moving population is 20–35%.^20–22^ In the TMJ, the temporomandibular disorder (TMD) affects up to 15% of adults, with a peak incidence at 20–40 years of age.^23^ In our previous study, we found that the spatio-temporal development of TMJ in pig embryos is similar to that in humans,^24^ and related studies also showed that pigs are ideal model animals for embryonic development studies.^25–29^ In this study, pigs were used as model animals to compare the spatio-temporal development characteristics of KNJ and TMJ, and to explore the different development characteristics of hyaline cartilage and fibrocartilage to provide the basis for joint disease pathogenesis, regenerative medicine and tissue engineering.

The joint development in the embryonic stage can be divided into three stages: the initial stage of joint development, the formation stage of each structure and the mature stage of joint development. The appearance of the mesenchymal condensate is the first sign of joint development.^30^ In this study, the E30 and E35 are the initial developments of KNJ and TMJ, respectively. The interzone including the two condensates can be seen in the KNJ. These two condensates divide into two parts along the axis of the tibia and femur, and they are connected with mesenchymal cells on E30. There is no patellar mesenchymal condensate formation on E30. Previous studies have shown that the patella is co-developed as part of the femur and gradually separates from the femur at a later stage of development.^31^ This result is consistent with previous study. Hip development begins on E30, with the formation of the mesenchymal condensate of the femoral head and acetabulum. The mesenchymal condensates of the femoral head and acetabulum are also formed. Hip development begins to cavitate and the joint cavity begins to appear. It may develop earlier than KNJ and TMJ. In the development of chondrocytes, the cartilage of the tibia and femur in the KNJ is already formed on E30. These chondrocytes are arranged in layers, and some cavitating chondrocytes can be seen among these chondrocytes. This result indicates that the chondrification of the femur and tibia began before E30 in pig embryos. In the human embryo, the chondrification of the femur and tibia begins at Carnegie stage (CS) 18(E44-E46).^32^ The chondrification time of femur and tibia in pig and human is similar (percentage of the whole pregnancy). In the development of TMJ, no formation of mesenchymal condensate was found on E30. However, there are two distant mesenchymal condensates formed on E35. The developmental origins of the KNJ and TMJ indicate that the TMJ develops from two mesenchymal condensates. Previous studies have shown that the KNJ is develops from one mesenchymal condensate.^33^^,^^34^ In this study, it was found that the KNJ originates from two interlinked mesenchymal condensate. This may be due to the fact that the one mesenchymal condensate is not yet completely separated. Pig KNJ development may have begun before E30. These two mesenchymal condensates are completely separated on E35. At this point, cracks in the joint formed by cavitation begin to form. This cavitation may be formed by the sequential action of apoptosis, microcavities, bone bending and elongation and HA accumulation.^35^^,^^36^ The study also showed that the embryonic development of the hip is earlier than that of the KNJ, which in turn is earlier than that of the TMJ. These results are in agreement with previous studies.

The formation phase of each structure in the KNJ and TMJ is from E45 to E55. The Knee tibial plateau, femoral condyle, patella, meniscus and cruciate ligament have been formed from E45 to E55. The chondrocytes of the femur and tibia of KNJ can be divided into four layers. The upper layer is the proliferative layer, and the cells are round, small and tightly arranged. The chondrocyte layer cells are round and oval in shape. Adjacent to the chondrocyte layer is a dense, columnar arrangement of oval group of chondrocytes. These chondrocytes are deeply stained with fuchsin O and develop into growth plates in the future. Previous studies have shown that articular cartilage cells and growth plate chondrocytes are derived from different progenitor cells.^37^ The lower layer of growth plate chondrocytes is a columnar arrangement of hypertrophic chondrocytes. The different ossification mechanisms of chondrocytes on both sides of growth plate need to be further studied. The KNJ has been cavitated and the joint cavity has been formed on E45. In contrast to KNJ, the superior and inferior articular cavities and the articular disk have been completely separated. The articular disk is thick at both ends and thin in the middle. In our previous study, it was shown that the inferior cavity was complete, while the superior cavity was not fully formed, being only a long narrow fissure.^24^ This result may be due to differences in the nutritional status of different litters and sows. In addition, the shape of the condyle is irregular and filled with a large number of chondrocytes. Compared with the KNJ, there was no growth plate chondrocyte formation under the condylar hypertrophic chondrocyte layer in TMJ. A large number of cartilaginous canals were found in the condylar chondrocytes to provide nutrients to the chondrocytes, but no significant cartilaginous canals were found in the KNJ until E55. These cartilaginous canals, located in the cartilage region, are loose vascular mesenchymal canals that assist in cartilage nutrition.^38^ They are formed by vascular endo-thelial growth factor (VEGF) stimulates the growth of vessels and the macrophages remove the cartilage matrix ahead the canals.^39^ When the embryo reaches 55 d of development, the growth plate of KNJ is more pronounced. The condyle, articular fossa and articular disk are complete formed. At this point, the KNJ structure enters a phase of gradual maturation.

From E75 to P30 is the mature stage of joint development. In the development of the KNJ from E75 to P90, the femur and tibia are continuously ossified. The growth plate is more pronounced. The cartilaginous canal in the chondrocyte region is gradually increases. During the maturation of the joint, it is noteworthy that on P0, the ossification of the femoral and tibial condyles occure from the center to the surrounding. The femoral and tibial condyles are ossified until P30. At this point, the growth plate is fully formed. There are regions of ossification on both sides of the growth plate. In contrast to the KNJ, there is no growing plate formation in the TMJ. The ossification of the condyle occurs below the chondrocyte layer, while the ossification of the KNJ condyle occurs at the center of the chondrocyte layer. Previous studies have shown that the mandibular condyle is the growth center of the mandible.^40^^,^^41^ It may perform a growth and development function similar to that of a growth plate.

The important developmental events of KMJ and TMJ in the embryonic development of miniature pigs are shown in Table 1. Although it has been shown that mesenchymal cells of the cartilage canal are the source of bone progenitor cells in the study of the mechanism of osteogenesis in plate.^38^ Whether there is a different mechanism of ossification between the upper and lower parts of the embryonic growth plate during the embryonic stage. Are there different molecular regulatory mechanisms between TMJ and KNJ? These questions are the focus of our next study.

**Table 1.** The important events during the embryonic development of KNJ and TMJ in this study.

Gestational/d age	KNJ	TMJ
E30	Interzone, mesenchymal condensate, femur and tibia chondrification, anterior tibial muscle, popliteal muscle and soleus muscle	No mesenchymal condensate
E35	Separation of mesenchymal condensate, joint cavity cavitation	Mesenchymal condensates, chondrification of temporal primordium, chondrocyte cavitation and Meckel’s cartilage
E45	Patella, meniscus, growth plate, articular cavity, suprapatellar bursa, patellar ligament, ossification of femoral shaft and tibial shaft	Superior articular cavity, inferior articular cavity, articular disk, cartilaginous canal and ossification of temporal bone
E55	Cartilaginous canal, cruciate ligament, infrapatellar fat pad	Condylar ossification, widening of superior and inferior articular cavities,
E75 and E90	Each structure gradually mature, crisscross arrangement of cartilage canals	Each structure gradually mature cartilage canals arranged along the longitudinal axis of the condyle.
P0	Ossification of epiphysis	Ossification of mandibular condyle, chondrocyte layer of condyle becomes thinner.
P30	Small cartilage canals in the epiphysis of femur and tibia	Mandible condylar process rich in cartilage canal

This is a preliminary comparative study of KNJ and TMJ in embryonic pigs. There are some important limitations in this study. The first deficiency is that each embryonic-age specimen is from a single sow litter. The development of KNJ and TMJ in different litters of miniature pigs may be related to the size, nutritional status, parity and litter size of the sows. The second deficiency is that only sagittal section analysis was performed, but not coronal section analysis. We believe that further studies on the development of KNJ and TMJ in pig embryos will advance research related to these two joints and subsequently improve the treatment of the disease.

## Conclusions

The KNJ develops earlier than the TMJ, with the growth plate being the center of development in the KNJ, but there is no growth plate in the TMJ, and the condyle of the TMJ is the center of its development. It is believed that with further research on the molecular regulatory mechanisms of KNJ and TMJ development in the embryonic stage, the treatment of joint-related diseases will be facilitated.

## Data and model availability statement

None of the data were deposited in an official repository. The data that support the study findings are available upon request to the corresponding author.
